# Investigation of the therapeutic applications, neuroanatomical targets and emerging technologies in deep brain stimulation surgery

**DOI:** 10.1186/1753-6561-9-S1-A39

**Published:** 2015-01-14

**Authors:** M Yostos, K Murphy

**Affiliations:** 1 RCSI Summer Research Student at the United Health Network (UHN) Toronto Western Hospital (TWH), Dept. of Radiology & Medical, Toronto, Canada; 2 Joint Department of Medical Imaging UHN Mt Sinai WCH Toronto, ON, M5G 2C4, Canada

## Background

First developed in France in 1987, Deep Brain Stimulation (DBS) is a neurosurgical intervention that involves the implantation of a brain pacemaker, which electrically stimulates specific brain nuclei.1 The FDA approved DBS for treatment for Essential Tremor in 1997, Parkinson’s Disease in 2002 and Dystonia in 2003.1 More recently, DBS has been indicated in management of neuropsychiatric disorders such as: Chronic Pain, Major Depression, Obsessive Compulsive Disorder and Anorexia Nervosa. Although effective, the exact mode of function of DBS remains poorly understood. Up to 2012, literature search yielded 84 peer-reviewed DBS studies that included over 296 psychiatric patients.2 The Canadian United Health Network (UHN) Krembil Neuroscience Centre is pioneering DBS research. Current neuroimaging, intra-operative electrophysiology and emerging DBS electrode and targeting technologies have improved DBS accuracy, effectiveness and acceptability. Objective: To examine the role of DBS in the management of motor and neuropsychiatric disorders and to provide, for the first time, a review of all anatomical DBS targets to date. To evaluate the accuracy of emerging DBS targeting and electrode technologies.

## Methods

Research for this paper was carried out using Pubmed, UHN online library, Medline, Google Scholar and UHN Grant Reviews. Papers were limited to those published in English between the years 2000 and 2013. Research was conducted at the Department of Radiology at the Toronto Western Hospital, UHN.

## Results

More than 26 anatomical targets have been identified for the treatment of movement and neuropsychiatric disorders. There is yet to be a large longitudinal cohort study confirming DBS usefulness and safety. DBS decreases disease burden by providing immediate improvement in the quality of life. Cost-utility analysis reveals that DBS has a high Quality-Adjusted Life Year (QALY) score.3 New neuroimaging technologies are enhancing DBS preoperative neuroimaging planning, intraoperative neuroimaging guidance and post-operative neuroimaging follow-up. Anatomical brain atlases, combined MR/CT, electrophysiological databases, registered surgical targets of previous patients and integrated functional and anatomical references have cumulatively shown to increase DBS targeting accuracy.4 Emerging research aims at developing electrodes from non-metals such as Carbon, in order to prevent metal-induced field distortion and MR artefacts. Guide tubes and stylettes are being introduced to refine DBS accuracy.

## Conclusions

DBS is a promising technology for severe, chronic movement and neuropsychiatric disorders. Continuous efforts to increase DBS targeting accuracy, improve on current DBS equipment systems and identify new anatomical targets are currently internationally pursued.

